# Cellular Polyolefin Composites as Piezoelectric Materials: Properties and Applications

**DOI:** 10.3390/polym12112698

**Published:** 2020-11-16

**Authors:** Ewa Klimiec, Halina Kaczmarek, Bogusław Królikowski, Grzegorz Kołaszczyński

**Affiliations:** 1 Lukasiewicz Research Network—Institute of Microelectronics and Photonics, Zabłocie 39, 30-701 Kraków, Poland; eklimiec@ite.waw.pl (E.K.); kolaszczynski@ite.waw.pl (G.K.); 2 Department of Biomedical Chemistry and Polymers, Faculty of Chemistry, Nicolaus Copernicus University, Gagarina 7, 87-100 Toruń, Poland; 3 Łukasiewicz Research Network—Institute for Engineering of Polymer Materials and Dyes—Toruń Division, M. Skłodowskiej-Curie 55, 87-100 Toruń, Poland; boguslaw.krolikowski@impib.lukasiewicz.gov.pl

**Keywords:** piezoelectric composites, polypropylene, polyethylene, cellular structure, piezoelectric sensors, energy harvesters

## Abstract

Piezoelectric polymers characterized by flexibility are sought for applications in microelectronics, medicine, telecommunications, and everyday devices. The objective of this work was to obtain piezoelectric polymeric composites with a cellular structure and to evaluate their usefulness in practice. Composites based on polyolefins (isotactic-polypropylene and polyethylene) with the addition of aluminosilicate fillers were manufactured by extrusion, and then polarized in a constant electric field at 100 V/µm. The content of mineral fillers up to 10 wt% in the polymer matrix enhances its electric stability and mechanical strength. The value of the piezoelectric coefficient d_33_ attained ~150 pC/N in the range of lower stresses and ~80 pC/N in the range of higher stresses, i.e., at ~120 kPa. The materials exhibited high durability in time, therefore, they can be used as transducers of mechanical energy of the human motion into electric energy. It was demonstrated that one shoe insert generates an energy of 1.1 mJ after a person walks for 300 s. The miniaturized integrated circuits based on polyolefin composites may be applied for the power supply of portable electronics. Due to their high sensitivity, they can be recommended for measuring the blood pulse.

## 1. Introduction

One of the challenges of contemporary technology is obtaining piezoelectric materials based on readily available polymers for the production of biomedical, portable sensors. The required non-toxicity, flexibility, low weight, easy surface shaping, any foil thickness, and the possibility of making wireless sensors work without the need for a power supply are the main reasons for the search of such materials. Recent studies show that these requirements can be met by piezopolymers, which in many cases could successfully replace ceramic piezoelectrics which are difficult to form [1,2]. The most known and applied material of this kind is poly(vinylidene fluoride), PVDF, as well as its copolymers [3,4,5]. This crystalline polymer has fluorine atoms with the highest electronegativity, and thus, highly polarized C–F bonds in each repeating unit: –(CF_2_–CF_2_)_n_–. Therefore, its piezoelectric properties are due to the permanent dipoles included in crystalline phases. PVDF occurs in several polymorphic forms with different properties, in which the β phase shows the highest dipole moment. Nowadays, this is used in highly professional applications [5,6,7,8,9,10,11,12,13].

The technology of acquiring smart devices and piezoelectric fabrics based on PVDF has been dynamically developing lately [7,8]. In earlier reports, the achievable power obtained from devices based on PVDF fibers ranged from a few nanowatts to microwatts, with higher values obtained for PVDF modified with inorganic piezoelectric substances, e.g., lead zirconate titanate. Recently, an efficient energy generator has been proposed using triaxial braided fibers of PVDF obtained by a modified manufacturing technology [7]. During compression or bending of such yarn, an output voltage of 380 mV and power density about 30 μW/cm^3^ can be achieved.

The latest report states that high-performance piezofibers from PVDF/BT (barium titanate) nanocomposites, produced in the melt-spinning process, have an output voltage of 4 V and power density of 87 μW/cm^3^. Therefore, they are recommended for self-powered wireless movement sensors, as well as wearable and portable energy harvesters [8].

The development of technology for the production of polymer piezoelectric sensors is very important from the point of view of medical applications, particularly in non-invasive impedance cardiography [10]. Thanks to the flexibility of polymeric materials, the sensor in the form of a comfortable wristband allows you to permanently monitor the work of the heart and assess the risk of dangerous consequences of cardiovascular diseases in patients staying at home.

The original research which concerned ultrasonic transducers shaped in a logarithmic spiral (Archimedean and Fibonacci) has just been published [9]. The authors of this idea designed their device as inspired by an echolocating skill of bats and dolphins. The performance of PVDF spirals, resembling the geometry of a snail, was investigated at multiple frequencies in a broad frequency range (20–80 kHz). The emission and receiving ultrasound signals were observed in all directions, therefore, this material was proposed in the application for sonar systems.

In addition, an intensive research into PVDF is related not only to its the piezoelectric properties. It is also a valuable material, especially in a modified form, for the production of membranes used, e.g., for fuel cells [11,12,13].

**Figure 1:**
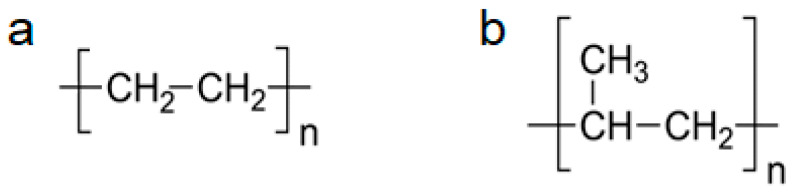
Chemical structure of (**a**) polyethylene and (**b**) polypropylene.

Nonetheless, the contribution of other readily available polymers to the production of piezoelectric devices is relatively small. Based on our previous research, we conclude that polyolefins such as isotactic-polypropylene (i-PP) or polyethylene (PE) of different types can fill this gap [14,15,16,17,18]. Both polymers, the structure shown in Figure 1a,b, are widely used and very cheap. Moreover, their processing is well known and uncomplicated. Between the carbon and hydrogen atoms, which almost do not differ in electronegativity, only covalent bonds appear. Therefore, these materials are non-polar.

However, only polymers showing the constant internal electric field may form electrets. In macromolecular compounds containing polarized chemical bonds, it is caused by the electric dipole moments in functional groups. In non-polar ones, it comes from the ordering of the free charge introduced to the material in the modification process. Structural defects or contaminations can also be a source of partial charging in macromolecules. A very effective method of creating electrets is the poling process, i.e., a charge injection during the polarization of polymeric specimen in a high electric field and elevated temperature. In the initial semi-crystalline polymer, the crystallites are randomly distributed in the amorphous phase, but they re-orient during the poling process, which changes the distribution of their charges and leads to polarization. The research showed that to keep the stability of electrets obtained this way, the polarization must be conducted in an electric field over 40 V/µm [19].

Many factors affect the piezoelectric properties of polymers, including chemical structure, molecular arrangement, specific interactions, internal morphology, polarizability, dielectric constant, mechanical properties, presence of additives, etc. Recently, special attention has been paid to polymers containing air/gas voids (also called cavities or cells) in the material bulk [20,21,22,23,24,25,26]. A high intensity electric field causes the microstorms described as silent or partial discharges even in completely non-polar polymers of cellular structure [26]. During such microdischarges, gas molecules from the air contained in the pores undergo ionization, resulting in the formation of both positive and negative charges, which are then located on the opposite walls of the cavities. In polarized cellular polymers, high internal stresses occur even under the influence of low deformation forces, and thus, the dipole moment can be significantly changed. As a result, high values of the piezoelectric charge may be obtained [23,24,25,26]. There are several possibilities of manufacturing cellular polymers [27,28,29,30,31,32,33]. One of the methods is the blow extrusion by gas injection into the polymer melt or double gas expansion presented in numerous references. In addition to piezoelectricity, foamed polymers have exceptional insulating properties, very low density, good mechanical properties, and the ability to damp vibrations.

Moreover, polymer doping with various inorganic or organic compounds, including nanofillers, brings an expected improvement in piezoelectric properties, i.e., an increase in the piezoelectric coefficient and enhancement of the durability of electrets. The presence of the mineral filler in the polymer matrix changes the molecular interactions, leads to disturbances in the local electric field, and simultaneously contributes to a change of internal morphology. The properly selected filler may contribute to the formation of the desired cellular structure. For instance, calcium carbonate (CaCO_3_) introduced to an extruded polymeric film causes the voids formation [30,32]. Other additional substances such as sodium bicarbonate (NaHCO_3_) or citric acid (C_6_H_8_O_7_), which decompose at elevated temperatures (60 and 153 °C, respectively), contribute to the formation of CO_2_-filled caverns [31]. Additionally, fillers play the role of nucleating agents, which lead to an increase in the degree of crystallinity of polymers, often resulting in improved mechanical properties and thermal stability. It has been found that the gas bubbles are created close to the structural defects and introduced filler nanoparticles [32,33].

The orientation process, i.e., stretching of the polymer film at room or elevated temperature [34] also has a significant impact on the properties of materials, including electrical ones.

The uniaxial or biaxial orientation of film influences the enhancement of air voids and increases the piezoelectric coefficient (d_33_) [22,33,35,36]. In addition, the shape of air or gas voids affects the piezoelectric properties, which is confirmed by experimental studies [22,31,33]. The cavities with an ellipsoidal shape, formed during the orientation process, led to a higher d_33_ value of the material. The open porosity of the film and the modification of the polymer surface causes an increase in the accumulation of surface charge and its more permanent entrapment [37].

The purpose of this work is to present useful piezoelectric properties of composites made of different types of polyolefins: Isotactic polypropylene (i-PP), high density polyethylene (HDPE), and medium density polyethylene (MDPE), as well as two aluminosilicate mineral fillers (Sillikolloid or glass beads). The article presents the piezoelectric properties of the best chosen materials suitable for the production of biomedical sensors, whose prototypes were made. The selection was done from numerous tested polyolefin composites (more than 40 systems with various aluminosilicate fillers and chalk), which were the subject of long-term research on piezoelectric polymeric systems, the physicochemical properties which have been published previously [14,15,16,17,18]. The obtained electrets were stored for a long time (up to 48 months) systematically determining their piezoelectric coefficient (d_33_). The focus was on systems that not only show favorable piezoelectric properties (high d_33_ values), but are also characterized by the best long-term stability over time. Our goal was to prove that these materials are suitable for the production of mechanical to electrical energy converters, and therefore, can be used for the production of energy harvesters or pressure sensors for general use.

## 2. Materials and Methods

### 2.1. Materials

The following polymers have been used for preparing composite films: Isotactic polypropylene i-PP Moplen HP456J type was produced by Basell Orlen Polyolefins, Płock, Poland, density of 0.91 g/cm^3^, the degree of crystallization was from 55 to ~65%; high density polyethylene HDPE Tipelin FS 471-02 type was produced by MOL Petrochemicals Co. Ltd., Hungary, density of 0.95 g/cm^3^, the degree of crystallization was ~60%; medium density polyethylene MDPE Tipelin FS 383-03 type was produced by MOL Petrochemicals Co. Ltd. (Tiszaújváros, Hungary), density of 0.94 g/cm^3^, the degree of crystallization was ~55%.

The fillers were two minerals: (a) Sillikolloid P87 was produced by Hoffmann Mineral (Neuburg, Germany) and consists of 35 wt% kaolin (Al_2_O_3_·2SiO_2_·2H_2_O), 55 wt% crystalline silica (SiO_2_), and 10 wt% colloidal silica; density of 2.60 g/cm^3^; particle size: Approx. from 1.5 to 10 μm with a high electric insulation resistance; (b) glass beads MinTron 7 was produced by RockTron, GB, with particle diameter < 30 μm; chemical composition of SiO_2_ 48–60%, Al_2_O_3_ 20–30%, and Fe_2_O_3_ 3–7%; alkali oxides 5–9%; and density of 2.20 g/cm^3^.

### 2.2. Preparation of Polyolefin Composites

Polyolefin (PO) in granulated form was mixed with a filler powder and the mixture was subjected to further processing. The composites containing 2.5, 5, or 10 wt% of the filler and neat polymers as reference samples were prepared by extrusion in a co-rotating twin-screw BTSK 20/40D laboratory extruder (Bühler GmbH, Braunschweig, Germany) in the following conditions: Extruder heating zones of 190, 195, 195, 190 °C, temperature of extrusion die head of 185 °C, and screw speed of 250 rpm. Composites obtained in the form of granules were then subjected to a further “cast” extrusion to get the ribbon of 145 mm wide and 100–120 μm thick using a single-screw Plasti-Corder PLV 151 laboratory extruder (Brabender OHG, Duisburg, Germany) in the following conditions: Extruder heating zones of 225, 235, 235 °C, temperature of extrusion die head of 245 °C, and screw speed of 75 rpm. Part of the films were further subjected to an uniaxial orientation, i.e., stretching at 3:1 ratio at 140 °C. The orientation reduced the film thickness by approx. half.

### 2.3. Composites Polarization

The polarization process has been carried out using a high voltage source (HVS) climatic chamber from HEAREUS-VÖTSCH (Hanau, Germany). The samples were placed between two metal contact electrodes and then heated up to a determined temperature (85 ± 2 °C). After reaching the upper temperature, the high voltage power supply was switched on successively enhancing the voltage value (in the range of 4.5 to 10 kV). The direct current was used.

The lower electrode was grounded. The electric field voltage, temperature, and time of polarization were selected experimentally avoiding an electric breakdown and taking into account the influence of polarization time on the values of piezoelectric coefficients. The optimal voltage was 100 V/µm with a temperature of 85 °C and time of 1 h. The polarization over 1 h did not affect the value of the charge coefficient d_33_.

### 2.4. Characterization of Composites Morphology and Physical Properties

Scanning electron microscope SEM 1430 (LEO Electron Microscopy Ltd., Cambridge, UK) has been used to study the composites morphology. SEM pictures were made after a brittle fracture of the samples in liquid nitrogen. Samples for SEM were sputtered with gold.

X-ray diffraction testing were carried out with a X’PERT Pro Philips Diffractometer (PANalytical B.V., Almelo, The Netherlands); Cu Kα1, wavelength 1.54056 Å, range 2θ 5°–90°, scan step size 0.020°, time per step 3 s). The mathematical distribution of the complex pattern into components has been done using the Voigt function to get the best fit of the experimental XRD shape.

The study of the mechanical properties were performed using a tensile testing machine type TIRAtest 27025 (TIRA Maschinenbau GmbH, Schalkau, Germany) at standard conditions (at room temperature, RT). Young’s modulus (E, MPa) was determined from the slope of the initial, rectilinear section of the stress-strain curve, with a proportional relationship between the strain and the tensile force. All results are the average of at least six measurements.

### 2.5. Characterization of Piezoelectric Properties

The piezoelectric coefficient d_33_ has been determined from measurements of the piezoelectric signal as a function of sample distorting force acting in 33 directions (where the direction of electric polarization is the same as the direction of the stress applied). The surface of the film samples in this case is 10 cm^2^. The measuring system consists of an electromagnetic actuator (ITE, Cracow, Poland), generator type Tektronix AWG 420 (Arbitrary Waveform Generator, Electronic Test Equipment, Cary, NC, USA), power amplifier P334 (Meratronik, Warsaw, Poland), tensometric load cell XLF 212R (Measurement Specialties, Inc., international company), amplifier ADR 154 (FGP Sensors Inc., France), Keithley’s electrometer 6517A (Keithley Instruments, Cleveland, OH, USA), and LeCroy’s oscilloscope LT-341 (LeCroy, Chestnut Ridge, NY, USA).

To execute the film distorting force, an electromagnetic actuator was powered by an amplified voltage being formed in the generator. The quantity and shape of the force course in time has been read and registered by the tensometric sensor, amplifier, and oscilloscope. For testing the force course, it has been set as a triangular shape, with an amplitude up to 150 N and a period of 1 s. The electric response of piezoelectric film to mechanic stress is transferred to the electrometer and oscilloscope. The configuration of the electrometer enables the charge and voltage measurements. The course of the acting force and the piezoelectric signal are simultaneously displayed on the oscilloscope. The obtained digital data enable further analysis. The film samples are placed between the stiff metal contact electrodes of 10 cm^2^ with a thickness of 3 mm to keep the proper tension distribution in the tested material.

The depolarization process of electrets was carried on a placing film sample between two metal contact electrodes lying on the Teflon bearers separating them from the heater. The whole system was insulated and screened from outside conditions. The heating speed was 0.018 °C/s, the temperature registration was from RT to 140 °C, and the arising TSD currents were registered using a multimeter 2002, electrometer 6517A, and visual basic application (VBA) script.

## 3. Results

**Figure 2:**
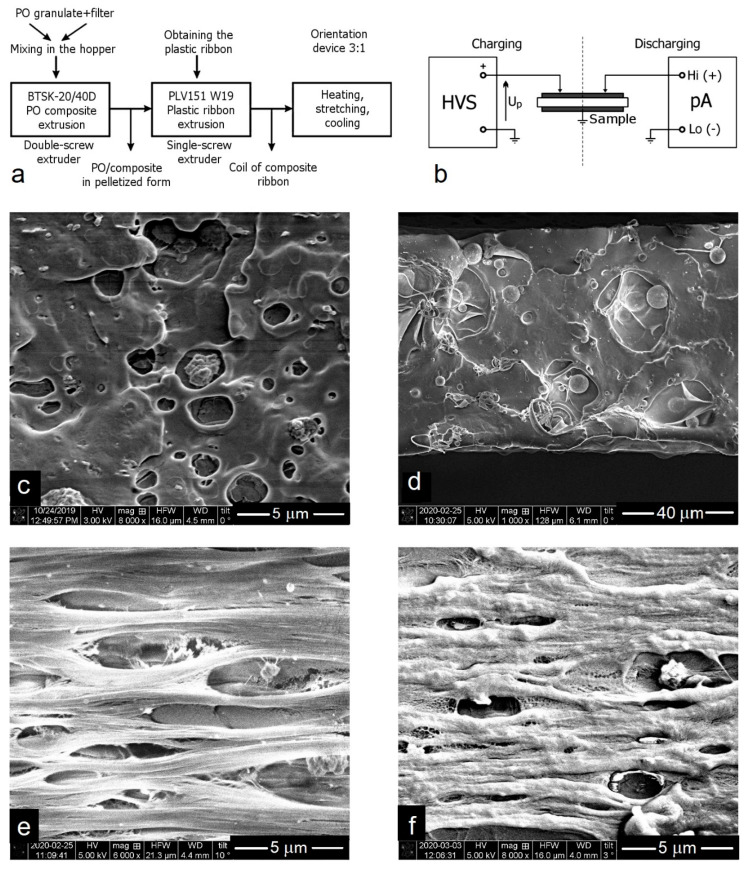
(**a**) Diagram of the technological process of composite manufacturing; (**b**) scheme of the film polarization and depolarization process; (**c**–**f**) representative SEM images of the selected oriented and non-oriented composite films (internal morphology visualized by a brittle fracture of film): (**c**) I-PP with 10 wt% of P87, non-oriented, (**d**) i-PP with 10 wt% glass beads, non-oriented, (**e**) i-PP with 5 wt% P87, oriented, (**f**) HDPE with 5 wt% P87, oriented.

The film of piezoelectric composites was obtained by extrusion in a two-step process in carefully selected conditions, which ensured good distribution of the filler in the polymer matrix and the reproducibility of the demanded properties. Part of the polymer films was further subjected to uniaxial orientation. This process causes stretching and ordering of randomly arranged macromolecules where the crystallites are dispersed. The most advantageous drawing ration is 3:1. The lower degree of stretching (2:1) did not significantly change the polymer crystallinity, but the higher one (4:1) resulted in the formation of microcracks or even film breaking. The diagram of the technological process of composite fabrication is presented in Figure 2a, while Figure 2b shows a scheme of device for the polarization/depolarization of samples. The chosen polarization temperature (85 °C) is higher than the temperature of glass transition (T_g_) of the polymer, but lower than the melting temperature (T_t_) of the crystalline phase. This allows for a sufficiently high mobility of non-ordered chains, in which the crystallites and molecular dipoles can be reoriented. Cooling the sample to room temperature freezes the induced dipoles, which leads to a permanent polarization and electret formation. It concerns both polymers: PP and PE. It should be emphasized that both cavities and filler particles are electric charge traps.

SEM images of cross-sections of polyolefin composites exhibit a typical heterogeneous inner structure. Filler particles of various sizes (from nanometric to larger agglomerates of the order of micrometers) are dispersed in the polymer matrix. There are also numerous, mostly spherical holes which are “empty” or partially occupied by filler particles. Such a structure is characteristic for all non-oriented composite films (Figure 2c,d). The orientation process causes stretching of the film and more parallel ordering of the fibrils in PP and PE, which is also associated with an increase in the degree of crystallinity of these samples. At the same time, cavities are elongated and take an ellipsoidal shape in the oriented samples, an example which is shown in Figure 2e,f. According to the literature, such shape of cells in the polymer bulk favors the piezoelectric properties [26].

Young’s modulus (E), which represents the stiffness of the specimen, was chosen to compare the mechanical properties of samples with different compositions. Generally, the 5–10% of the introduced filler contributes to some increase in E compared to neat polymers, but the orientation of composite films has a greater, positive effect on this mechanical parameter.

In Table 1, Young’s modulus is presented only for the best piezoelectric materials obtained (the results for the remaining tested samples can be found in previously published works [14,15,16,17,18]). It has been found that the oriented i-PP composite containing 5% Sillikolloid P87, which showed the highest Young’s modulus of ~1900 MPa stands out among the tested samples. Moreover, in the case of non-oriented films, the i-PP with 10% Sillikolloid P87 or 10% glass beads exhibits high E values (1143 and 1259 MPa, respectively). Furthermore, considering HDPE-based composites, it can be concluded that the 10% addition of Sillikolloid P87 or glass beads contributes to high E values of over 1000 MPa (this applies to oriented films).

On the other hand, MDPE composites show the lowest mechanical properties. As can be seen from Table 1, the best mechanical properties among the samples based on this polyethylene show the oriented film with the addition of 5% Sillikolloid P87, where E attains 790 MPa.

It is difficult to explain the distinct differences between properties of composites based on HDPE and MDPE due to the fact that both polyolefins are built from the same repeating unit (–CH_2_-CH_2_–). However, as we know, even subtle differences in the structure of macromolecules can noticeably change their behavior, which was confirmed by means of infrared spectroscopy [18]. A detailed analysis of the FTIR spectra in the area of CH_2_ and CH_3_ deformation vibrations showed a slightly higher degree of MDPE branching compared to HDPE, which is also the reason for the different degrees of crystallinity of both these polymers.

**Figure 3:**
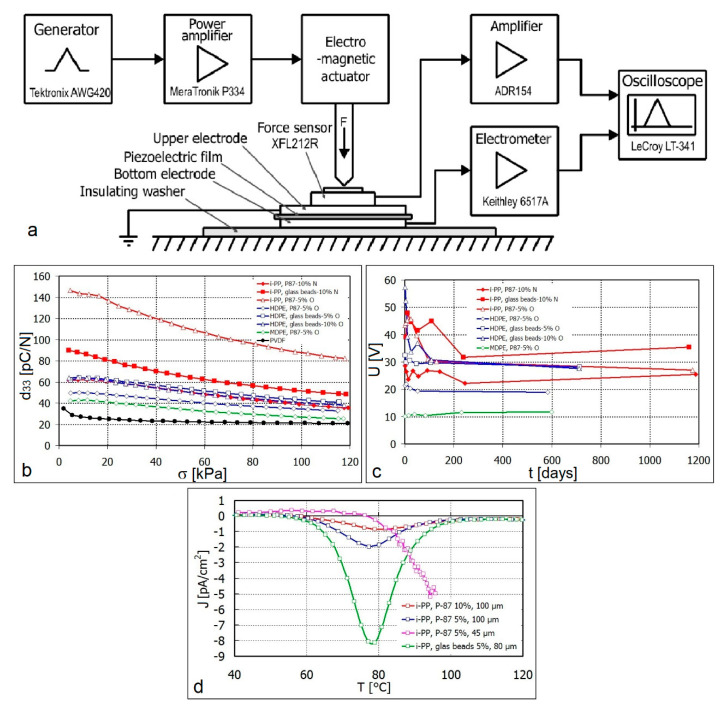
(**a**) Scheme of the device for measuring piezoelectric signals; (**b**) dependence of the d_33_ coefficient vs. mechanical stress; (**c**) dependence of the piezoelectric voltage on the storing time of electrets in room temperature (RT); (**d**) dependence of the density of depolarization currents (J) vs. temperature for the selected electrets.

The piezoelectric properties of the investigated composite films of cellular structure were determined by the d_33_ coefficient. The film distorting force acts perpendicularly to the sample surface and corresponds to the direction of the electric field in the electret. The scheme of the electric signal measuring system, depending on the stress force, is shown in Figure 3a.

The piezoelectric charge (Q) is proportional to the force (F) acting on the film sample, according to Equation (1):

Q = d_33_ × F (1)

where d_33_ is the piezoelectric coefficient.

Considering the sample surface (A), we get the relationship where the density of the piezoelectric charge q is proportional to the mechanical stress value σ:

q = d_33_ × σ (2)

where σ = F/A.

Therefore, the charge piezoelectric coefficient d_33_ (expressed in pC/N), which was used to compare the properties of different samples (Table 1), was calculated from the relationship as:

d_33_ = q/σ (3)

It should be noted that the piezoelectric coefficient is not an ordinary proportionality constant in Equation (2), since the relationship q = f (σ) is not rectilinear. Here, d_33_ generally decreases with the increasing stress, which has been documented many times [15,16,17,18]. Hence, a more accurate name for the parameter d_33_ is the piezoelectric coefficient.

The values of the measured piezoelectric coefficients d_33_ for the studied composites depending on the stress applied, are shown in Figure 3b and Table 1. As can be seen, the course of changes in d_33_ are typical for the polymeric piezoelectrets. The values of this parameter are higher for low stress (10-20 MPa) and drop with the increase of the applied stress (up to 120 kPa). The observed decrease is smaller, the smaller initial value of d_33_ is at low stresses.

The samples of oriented i-PP with 5% of Sillikolloid P87 and non-oriented i-PP containing 10% glass beads are characterized by exceptionally high values of d_33_ in the entire range of the stresses tested. The remaining samples, in which the d_33_ values are presented in Figure 3b, also show very good piezoelectric properties. Apart from i-PP with 10% of Sillikolloid P87, these are also oriented films of HDPE and MDPE based composites. These favorable properties of the tested composites result from the cellular structure discussed above, confirmed by SEM.

The durability of electrets was tested by keeping films between two compacted metal layers at room temperature to screen them from the outer electrostatic charge. The piezoelectric measurements were carried at a stress of 100 kPa. The results of the obtained piezoelectric voltage during the storage of samples through 1200 days are shown in Figure 3c. As can be seen, after the first period (several or several dozen days when the voltage and charge fluctuations are observed), the piezoelectric parameters stabilize. The stabilization time of the piezoelectric parameters of the polymer can be shortened by heating it to 60 °C for 30 min. The most promising samples showed a shelf life of up to 1200 days. These are i-PP based composites containing 5–10% of the filler.

Due to the very long depolarization process, the thermally stimulated discharge currents (TSDC) have been applied [38,39,40]. Regarding the direction of the polarizing voltage, the sample was placed in the same way, as shown in Figure 2b. This method can be used to determine the temperature range in which the depolarization process is very slow. The temperature T_m_ read at an extreme point of the curve is the temperature where the density of the depolarization current is highest and the material may be used only for a short time.

The TSDC investigation showed that the i-PP film with 10 wt% Sillikolloid P87 is more durable than the film with glass beads. It may work in a permanent way up to 60 °C and its T_m_ is ~80 °C. The i-PP film with glass beads has both temperatures lower by 10 °C, i.e., ~50 and ~70 °C, respectively. It can be assumed that in the i-PP sample with Silikolloid P87, the polymer partially intercalates between the kaolin plates of the filler during processing, which raises its operating temperature. For the oriented i-PP film with 5 wt% Sillikolloid P87, the permanent working temperature attains 70 °C and the temperature T_m_ over 90 °C. The orientation of films causes the air voids enhancement in the material and growth of the crystalline phase, therefore, elevating its thermal strength.

Composites based on HDPE and MDPE containing the Sillikolloid P87 filler have a lower d_33_ value, but they may work permanently up to ~90 °C and the T_m_ attains over 120 °C.

Taking into account the highest d_33_ value and range of film elasticity as well as their durability, the three best samples, the parameters which are marked in bold in Table 1, can be recommended for practical applications. It should be emphasized that all the presented samples have a much higher piezoelectric coefficient than PVDF (a polymer that can be a benchmark), the d_33_ which is in the range of 13–28 pC/N [41,42].

**Figure 4:**
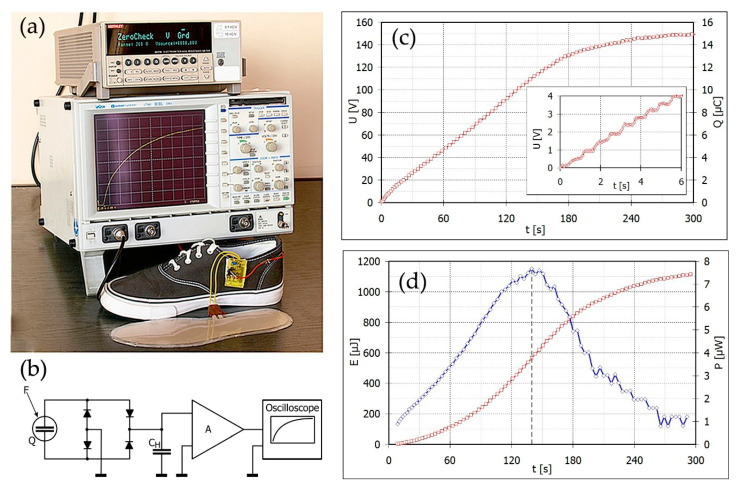
(**a**,**b**) General view and an electric scheme of the measuring set for the electric signal and mechanical electro-transformer as a shoe insert (Q: Source of piezoelectric charge, C_H_: Harvesting capacitor, A: Amplifier, F: Acting force), (**c**) speed and the mode of charging capacitor (the inset contains the enlarged part of the curve for the first 6 s), (**d**) dependence of energy and the capacitor’s power vs. walking time. Non-oriented film of i-PP with 10 wt% Sillikolloid P87 was used for the fabrication of the piezoelectric element.

In order to determine the practical usefulness of the studied composites as a converter of mechanical energy (from a human walk) into electric energy and its storage in a condenser (harvesting energy), the prototype of a shoe insole with a piezoelectric element based on filled i-PP was made (Figure 4). The non-oriented film of i-PP with 10 wt% Sillikolloid P87 showing the highest compression strength has been selected for this test and placed between two layers of the poly(ethylene terephthalate) film (PET). The internal surface of PET has been covered with silver electrodes by serigraphy. The shoe insert prepared in this way has been placed in the shoe sole. Under the pressure of the foot on the piezoelectric film, a voltage appears on the electrodes (Figure 4c). As a result, a capacitor with a capacity of C_H_ = 0.1 μF (the circuit shown in Figure 4b) is charged with each pressure (that is, with each step). The voltage has a variable polarity, therefore, in order to collect all the energy, a diode system was used (Szhottky diodes BAT 54) with a very low reverse current of a few pA. The rate of energy growth is shown in Figure 4d. To visualize the charging process, the measuring system was connected to the oscilloscope using an operational amplifier with a high input resistance of 1 TΩ, which does not interfere with the process. A man weighing 78 kg walked the treadmill at a speed of 4 km/h. The harvested energy (Eg) and the power (P) of the voltage source were determined from the following dependencies 4 and 5:

Eg = U^2^ · C/2 (4)

where U is the voltage and C is the capacity:

P = dEg/dt(5)

After 300 s of walking, the energy stored on the capacitor is equal to 1100 µJ, while the power of 7.5 µW is achieved just after 140 s (Figure 4d).

The second example of the studied composite application is the pressure sensor. It has been observed that in the case of the polymer film with mineral fillers characterized by a cellular structure, the use of contact electrodes is advantageous. During mechanical deformation, the electric charge is transferred to the electrode in an inductive manner. The depositing electrodes directly on the material causes a significant decrease in the value of the electrical signal, therefore, this solution was rejected here.

**Figure 5:**
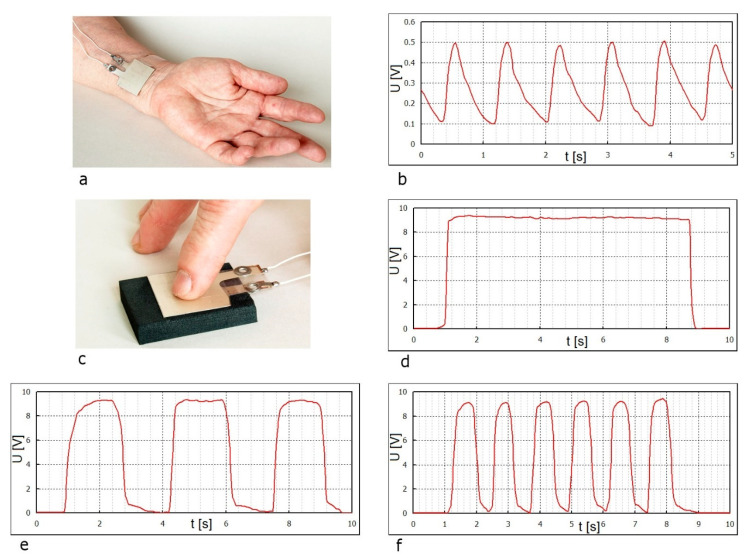
Example of the sensor application for medical use: Blood pulse measurement (**a**), dependence of the piezoelectric voltage arising from the artery pulsation (**b**), finger pressure measurement (**c**), piezoelectric voltage curves for variable finger pressure frequencies (**d**–**f**). The piezoelectric element was made of i-PP oriented film with 5 wt% Sillikolloid P87.

For pressure sensor fabrication, an oriented film i-PP with 5 wt% Sillikolloid P87 has been chosen. Figure 5a shows the method of placing a sensor on the human wrist, while the electric signals obtained for blood pulsation is presented in Figure 5b. The obtained voltage cyclically reaches 0.5 V every 1 s.

The second sensor, shown on the next photo (Figure 5c), has been examined for obtaining a current from the finger pressure. Plots of piezoelectric voltage as a function of force action time were recorded for a variable finger pressure frequency (Figure 5d–f). A light touch of a finger generates a voltage of approx. 10 V. It is caused by the changes of the dipole moment in the material, (i.e., increase of electric field) under the deformation force. The frequency of the voltage changes is consistent with the frequency of the deformation force. As we can see from Figure 5d, the piezoelectric signal at the load of the electric system of resistance 10^14^ Ω may be kept for a longer time. In this case, the pressure force is constant for 8 min, therefore, the tension is also constant.

Figure 5b,d–f indicates that the developed piezoelectric films are very sensitive and react immediately upon the application of a deforming force.

## 4. Final Discussion and Conclusions

The extrusion process and polarization in the constant electric field of intensity 100 V/μm at 85 °C allow creating piezoelectric composites of a cellular, heterogeneous structure consisting of polyolefin and mineral filler in a relatively simple and cheap way. The composition and manufacturing conditions have been optimized. There is no need to apply additional processing aids or compatibilizers.

**Figure 6:**
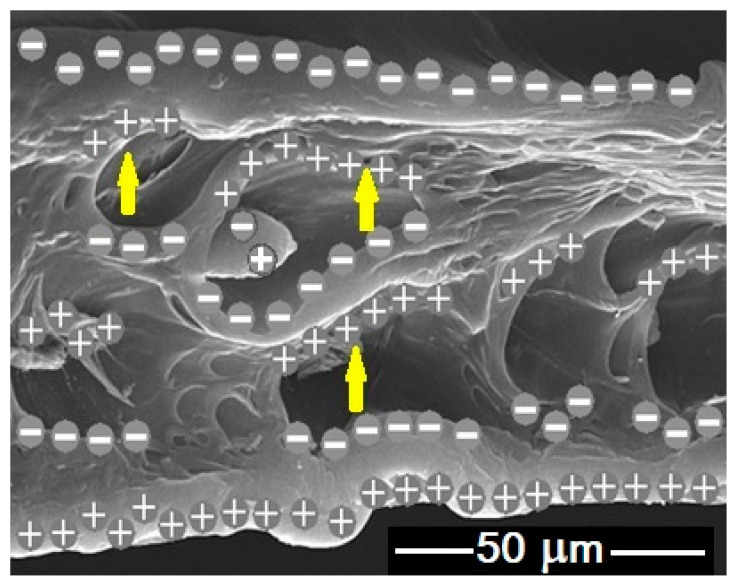
Schematic presentation of electrets in the cellular polyolefin composite based on an SEM image of the sample cross-section (yellow arrows show examples of dipole moment vectors occurring in the cavities).

A schematic representation of the polarized structure of the cellular, filled polymer is shown in Figure 6. Resulting from the poling process (starting with the ionization of gas in the material pores), an electric charge of opposite signs is located at the phase boundaries in polymer cells (cavities), thus, permanent dipoles necessary to induce a piezoelectric effect are created. Dipoles can also form at the interface between the crystalline and the amorphous phases of the polymer. The cavities in the polymer matrix are partially occupied by mineral filler particles. External compressive forces cause the distortion of structure and redistribution of electric charges, which can be monitored.

The advantage of manufacturing piezoelectric composites by extrusion includes the temperature not exceeding 200 °C, i.e., much lower than required in the processing of piezoelectric ceramics (usually higher than 1000 °C) and the long-term stability of piezoelectric properties during storage under ambient conditions (up to 40 months). It should be emphasized that in order to obtain reproducible piezoelectric properties, strictly defined conditions for obtaining, polarization, and composition of the presented materials must be followed.

The highest received value of the piezoelectric coefficient d_33_ was 150 pC/N for the i-PP non-oriented film with 5 wt% Sillikolloid P87, which was used for the preparation of a transducer of mechanical energy into electricity from a human walk. The energy obtained by walking a person in shoes with a piezoelectric insert for 300 s attained 1.1 mJ.

Such converters, the prototypes which are presented in this work, can supply energy to portable and wearable electronic devices. They are safe for humans as they do not contain toxic heavy metals such as inorganic piezoelectric materials. Therefore, their use for biosensors implanted into the body could also be suggested. Moreover, as stated, the sensors based on polyolefin composites are very sensitive and may register small moves of the human body, e.g., blood pulse or finger touch. Due to the low production costs, the availability of raw materials (both polyolefins and fillers), ease of processing, light weight, high flexibility, and good mechanical strength of the materials, they can be used for the production of universal sensors of any shape and size for common use. The designed and made sensor prototypes are the first stage leading to the implementation of the developed materials for industrial production.

## 5. Patent

Polish patent, PL 235140, 21.02.2020, B. Królikowski, H. Kaczmarek, E. Klimiec, Sposób wytwarzania folii polietylenowych o właściwościach piezoelektrycznych (a method of producing polyethylene films with piezoelectric properties).

## Figures and Tables

**Table 1:** Values of Young’s modulus (E) and piezoelectric coefficients (d_33_) for the selected composite films characterized by the best piezoelectric properties (O: Oriented, N: Non-oriented; the best piezoelectric composites and the highest values of the E and d_33_ parameters are signed in bold).

Composite		E [MPa]	d_33_ [pC/N]
**i-PP + 5 wt% P87**	O	**1893**	**150–80**
**i-PP + 10 wt% glass beads**	N	**1143**	**90–50**
**i-PP + 10 wt% P87**	N	**1259**	**60–40**
HDPE + 5 wt% glass beads	O	809	60–40
HDPE + 10 wt% glass beads	O	**1060**	60–40
HDPE + 10 wt% P87	O	**1166**	50-35
MDPE + 5 wt% P87	O	790	40–20
